# Association between Computed Tomography-Determined Loss of Muscle Mass and Impaired Three-Month Survival in Frail Older Adults with Cancer

**DOI:** 10.3390/cancers15133398

**Published:** 2023-06-28

**Authors:** Antti Tolonen, Hanna Kerminen, Kaisa Lehtomäki, Heini Huhtala, Maarit Bärlund, Pia Österlund, Otso Arponen

**Affiliations:** 1 Department of Radiology, Tampere University Hospital, Kuntokatu 2, 33520 Tampere, Finland; 2 Faculty of Medicine and Health Technology, Tampere University, Arvo Ylpön Katu 34, 33520 Tampere, Finlandpia.osterlund@helsinki.fi (P.Ö.); 3 Centre of Geriatrics, Tampere University Hospital, Kuntokatu 2, 33520 Tampere, Finland; 4 Gerontology Research Center (GEREC), Tampere University, Arvo Ylpön Katu 34, 33520 Tampere, Finland; 5 Department of Oncology, Tays Cancer Centre, Tampere University Hospital, Teiskontie 35, 33520 Tampere, Finland; 6 Faculty of Social Sciences, Tampere University, Kalevantie 5, 33014 Tampere, Finland; 7 Department of Oncology, Comprehensive Cancer Center, Helsinki University Hospital, University of Helsinki, Haartmaninkatu 4, 00290 Helsinki, Finland; 8 Department of Gastrointestinal Oncology, Tema Cancer, Karolinska Universitetssjukhuset, Eugeniavägen 3, 17176 Solna, Sweden; 9 Department of Oncology-Pathology, Karolinska Institutet, Solnavägen 1, 17177 Solna, Sweden

**Keywords:** computed tomography, loss of muscle mass, sarcopenia, cancer, frailty, older adults

## Abstract

**Simple Summary:**

A large proportion of older adults are not fit for oncological treatments due to frailty and comorbidities. To aid in the decision-making of whom to provide active oncological treatment to, we used G8-screening and comprehensive geriatric assessment in patients at risk of frailty. We studied the added value of muscle measurement with computed tomography (CT) at the third lumbar vertebra level in these potentially frail ≥ 75-year-olds. In 58 patients with advanced or metastatic solid tumors, a higher 3-month mortality rate and poorer nutritional status and functioning were noted among those with low muscle mass, independent of other predictive factors. Most patients with low muscle mass were allocated to best supportive care only. A poorer 2-year survival among 21 patients treated with curative intent was noted in those with low muscle mass. Muscle mass assessment alongside geriatric assessment can thus help oncologists identify patients at increased risk of severe toxicities and with little benefit from oncological treatments.

**Abstract:**

As patients with solid (non-hematological) cancers and a life expectancy of <3 months rarely benefit from oncological treatment, we examined whether the CT-determined loss of muscle mass is associated with an impaired 3-month overall survival (OS) in frail ≥75-year-old patients with cancer. Frailty was assessed with G8-screening and comprehensive geriatric assessment in older adults at risk of frailty. The L3-level skeletal (SMI) and psoas (PMI) muscle indexes were determined from routine CT scans. Established and optimized SMI and PMI cut-offs were used. In the non-curative treatment group (n = 58), 3-month OS rates for normal and low SMI were 95% and 64% (HR 9.28; 95% CI 1.2–71) and for PMI 88%, and 60%, respectively (HR 4.10; 1.3–13). A Cox multivariable 3-month OS model showed an HR of 10.7 (1.0–110) for low SMI, 2.34 (0.6–9.8) for ECOG performance status 3–4, 2.11 (0.5–8.6) for clinical frailty scale 5–9, and 0.57 (0.1–2.8) for males. The 24-month OS rates in the curative intent group (n = 21) were 91% and 38% for the normal and low SMI groups, respectively. In conclusion, CT-determined low muscle mass is independently associated with an impaired 3-month OS and, alongside geriatric assessment, could aid in oncological versus best supportive care decision-making in frail patients with non-curable cancers.

## 1. Introduction

As the population ages, the number of older adults with cancer increases [1]. Older adults with cancer present unique challenges to cancer care providers, such as an increased number of comorbidities, frailty, loss of physical and mental function, malnutrition, and muscle-wasting conditions (e.g., sarcopenia and cachexia) [2]. To achieve optimal treatment outcomes and adherence, the oncologist should take into account patients’ physical, mental, and sociopsychological resources, as well as tumor-related factors and treatment intent. Indeed, patients with a solid tumor and a short life expectancy (i.e., less than 3-month overall survival (OS)) probably do not benefit from oncological treatments and are often excluded from oncological studies [3,4].

Comprehensive geriatric assessment (CGA) for older adults with cancer is regarded as the gold standard in the identification of frailty and other vulnerability-associated conditions [5]. Indeed, compared to usual care, CGA-guided oncological treatment has been shown to reduce treatment-related toxicities [6,7] and treatment discontinuations [8]. However, CGA does not directly determine which patients could undergo oncological treatment, and there is heterogeneous evidence that CGA’s implementation improves survival outcomes [5,6,7,9]. Further tools are needed to supplement CGA to enable a better characterization of the phenomena underlying frailty and augment the assessment of treatability.

Sarcopenia is a syndrome often seen in older adults with cancer and in patients with other serious debilitating diseases. The syndrome is characterized by the progressive and generalized loss of skeletal muscle mass and strength [10]. Cachexia is a more complex condition with both “objective” components (e.g., inadequate food intake, weight loss, inactivity, loss of muscle mass, and metabolic derangements that induce catabolism) and “subjective” components (e.g., anorexia, early satiety, taste alterations, chronic nausea, distress, fatigue, and loss of concentration) and affects approximately half of patients with advanced cancers [11]. The diagnosis of sarcopenia requires low muscle strength combined with low muscle mass or quality [11], whereas the diagnosis of cachexia requires that phenotypical criteria (weight or muscle loss) and etiological criteria (systemic inflammation, reduced food intake) are met [11]. Sarcopenia (with skeletal muscle loss as a surrogate endpoint) in patients with cancer is associated with a decreased survival rate [12,13,14,15,16,17,18,19,20], an increased risk of oncological treatment toxicities [14,20,21,22], and an impaired health-related quality of life [23]. As diagnosing muscle-wasting conditions early is important for optimized treatment planning, complementary means to detect these conditions are of great interest.

Computed tomography (CT) is an accurate imaging modality for body composition analysis and enables the identification of skeletal muscle-wasting conditions [24]. The opportunistic use of CT scans to quantify the amount of muscle tissue based on Hounsfield units is often possible because patients with cancer are imaged with CT as part of their diagnostic work-up and during treatment to assess treatment response [24]. The most used level in body composition analysis is the third lumbar vertebra, from where the area of all the axially visible muscles or individual muscles (e.g., the psoas muscles) are measured [24]. Analogously to the body mass index (BMI), muscle areas are often normalized by the patient’s squared height resulting in muscle indexes, such as the skeletal and psoas muscle indexes (SMI and PMI, respectively). However, the role of these parameters remains poorly studied in older adult patients with cancer, especially those who are frail, for whom there are no established SMI or PMI cut-off values.

The primary aim of this study was to examine the association between CT-determined low muscle mass with published and optimized cut-offs and 3-month OS rates. The secondary aim was to investigate whether low muscle mass offers additional predictive value for treatment decisions in combination with oncological and geriatric evaluations in frail older adults with cancer.

## 2. Materials and Methods

### 2.1. Study Cohort

**Figure 1:**
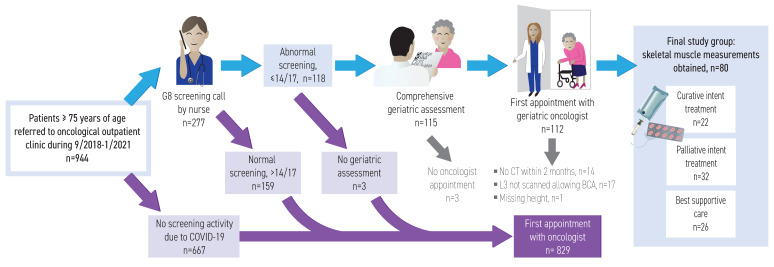
Flowchart for geriatric oncological patients at the Comprehensive Cancer Center of Tampere University Hospital.

The study cohort was collected retrospectively among patients referred to an oncology outpatient clinic for consideration of oncological treatment at the Cancer Center at Tampere University Hospital, Finland, from September 2018 to January 2021. According to protocol, an oncology nurse called the referred patients aged ≥75 years or their relatives to conduct G8-screening to identify patients at risk of frailty. The G8-screening tool consists of eight questions with a maximum of 17 points, with at-risk patients having ≤14 points [25]. Patients at risk of frailty were referred to a geriatrician for CGA before their appointment with an oncologist. Patients who received more than 14 points in the G8-screening underwent routine treatment without CGA and were not included in this study (flowchart in Figure 1).

The inclusion criteria for the patients were as follows: the patient had an abnormal G8-screening result and underwent geriatric and oncological evaluation at the Geriatric Oncological Unit; the patient’s clinical information was available; and the patient had an appropriate CT scan. The scan had to fulfill the following criteria: the midpoint of the third lumbar vertebra was scanned in a manner that allowed body composition analysis; the scan was performed no more than 2 months prior to CGA according to local standard practice [26] (whereas a maximum of 1 month is frequently used in study populations [27]); and the scan was performed before starting any systemic oncological treatment.

### 2.2. Ethics Statement

The study was approved by the local institutional review board at Tampere University Hospital (study numbers R19628S and R20503S). Ethics Committee approval and written informed consent are not needed in single-institution register-based studies in Finland.

### 2.3. Body Composition Analysis

**Figure 2:**
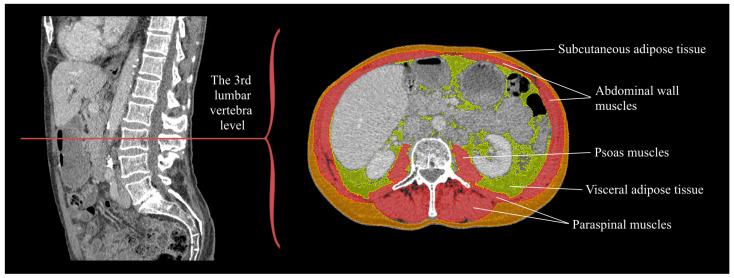
Body composition analysis is performed from an axial slice at the midpoint of the third lumbar vertebral level. The patient had a low optimized skeletal muscle index (SMI) value of 45.7 cm^2^/m^2^ and an optimized psoas muscle index (PMI) of 5.06 cm^2^/m^2^.

Appropriate CT studies were collected from the hospital’s Picture Archiving and Communication Systems (Commit; RIS IDS7 Radiology Desktop, Sectra AB, Linköping, Sweden). One reader (A.T.) performed body composition analysis with the 3D Slicer software (version 4.11) [28]. A single axial slice was used from the midpoint of the third lumbar vertebra level, determined using the sagittal and coronal images. One patient with incomplete abdominal muscle scanning was excluded from SMI analysis. Slice volumes were divided by slice thicknesses (0.45–3.00 mm) to calculate areas. The Hounsfield unit range for skeletal muscle tissue was set to −29 to 150 [29] and used irrespective of contrast agent use and scan phase. Manual correction of the tissues was performed according to morphology and/or Hounsfield unit variability. SMI and PMI values were calculated by dividing the whole skeletal muscle and psoas areas, respectively, with the patient’s squared height (Figure 2).

### 2.4. Clinical Data Acquisition

Patients’ age, sex, dates for the CT scan, visits to the geriatrician and the oncologist, and date of death if deceased were collected from the hospital’s electronic patient records. The primary tumor site and extent (local vs. locally advanced/metastatic) were recorded. The Eastern cooperative oncology group performance status (ECOG PS) was recorded at the oncologist’s appointment [30]; the FRAIL scale [31], clinical frailty scale [32], activities of daily living, instrumental activities of daily living [33], hand-grip strength, and sit-to-stand tests [34] were recorded by the geriatrician at the CGA. Nutritional status was assessed with the mini nutritional assessment-short form [35] and with the Global Leadership Initiative on Malnutrition (GLIM) classification [36]. For GLIM, phenotypic criteria included weight loss of >5% per 6 months, BMI < 22 kg/m^2^, or impaired hand-grip strength with cut-offs as in [34]; we excluded reduced muscle mass as a criterion because there were no quantitative muscle mass records in the patient database. Etiologic criteria were serum albumin < 35 g/L or C-reactive protein > 10 mg/L [37]. Hemoglobin < 110 g/L, creatinine > 100 µmol/L for men and >90 µmol/L for women, and albumin < 30 g/L were also recorded [38].

The patients were categorized into curative and non-curative intent treatment groups. Patients in the non-curative treatment group were further subdivided into the palliative chemotherapy group and the best supportive care only group (BSC).

### 2.5. Statistical Analyses

The association between CT-determined low muscle mass and 3-month OS was studied with Kaplan–Meier estimation and Cox regression models. The 3-month OS was calculated from the oncologist’s visit until the end of the 3-month follow-up or death. The cut-offs for low muscle mass were optimized stepwise. We first used SMI [12,39,40,41,42] and PMI [43,44] cut-off values from the literature to examine their performance in our study cohort. As these cut-offs were not optimized for frail older adults with cancer, we also tested sex-specific medians and receiver operating characteristic (ROC)-determined optimized cut-offs. To optimize the SMI and PMI cut-off values, Youden’s indexes (sensitivity + specificity − 1) were calculated, and the highest Youden’s index was chosen, giving sensitivity and specificity equal weight.

Patient demographics are presented as absolute values and percentages and as medians with ranges or interquartile ranges (IQRs), and muscle index parameters are presented as means with standard deviations. Logistic regression and odds ratios (ORs) with 95% confidence intervals (CIs) were used to examine differences between the muscle indexes and patient characteristics. The OS for the whole follow-up period was calculated from the oncologist’s visit until the end of January 2022 or death. Established factors that predict OS (ECOG PS, clinical frailty scale) and sex due to the difference in muscle mass distributions were included in multivariable analyses.

All *p*-values were two-sided and considered significant when *p* ≤ 0.05, without adjustment for multiple analyses. Data were analyzed with SPSS (IBM SPSS Statistics for Windows, 2020, Version 27.0.0.1, IBM Corporation, Armonk, NY, USA).

## 3. Results

### 3.1. Patient Demographics

A total of 80 patients (43 men and 37 women) were included with a median age of 80 (range 75–91) years (Figure 1). The median time between the CT scan and the oncologist visit was 21 (range 0–61) days. The cohort consisted of patients with upper GI, lower GI, and other cancers (Table 1, with details in Table S1).

### 3.2. Initiated Treatments

All patients treated with curative intent (n = 22; 28%) underwent surgical resection. At the time of the oncologist’s appointment 20/22 (91%) had been operated and were considered for adjuvant chemotherapy and 2/22 (9%) were considered for neoadjuvant treatment. All patients in the curative intent treatment group receiving curative oncological treatments (n = 10) were given chemotherapy.

Treatment intent was non-curative in 58 (73%) patients; 31 received palliative oncological treatment and 27 BSC only. Of the 58 patients in non-curative care, 45 had metastatic disease and 13 had locally advanced disease ineligible for curative surgery because of disease extent, frailty, or poor functional status. The palliative oncological treatments (n = 31) that were given were chemotherapy (n = 18), chemotherapy and bevacizumab (n = 3), hormonal treatment (n = 2), hormonal treatment with denosumab or radionucleotides (n = 4), tyrosine kinase inhibitors (n = 3), and immuno-oncologic treatment (n = 1). Six patients were given radiotherapy in combination with other treatments.

Having a lower GI cancer was associated with curative treatment intent but there were no other statistically significant differences in baseline characteristics between the curative and non-curative treatment groups (Table 1 and Table S1).

### 3.3. Muscle Index Cut-Offs

The mean SMI values for men and women were 43.3 ± 6.1 cm^2^/m^2^ and 35.7 ± 4.6 cm^2^/m^2^, and mean PMI values were 5.30 ± 1.04 cm^2^/m^2^ and 4.34 ± 1.04 cm^2^/m^2^, respectively.

**Figure 3:**
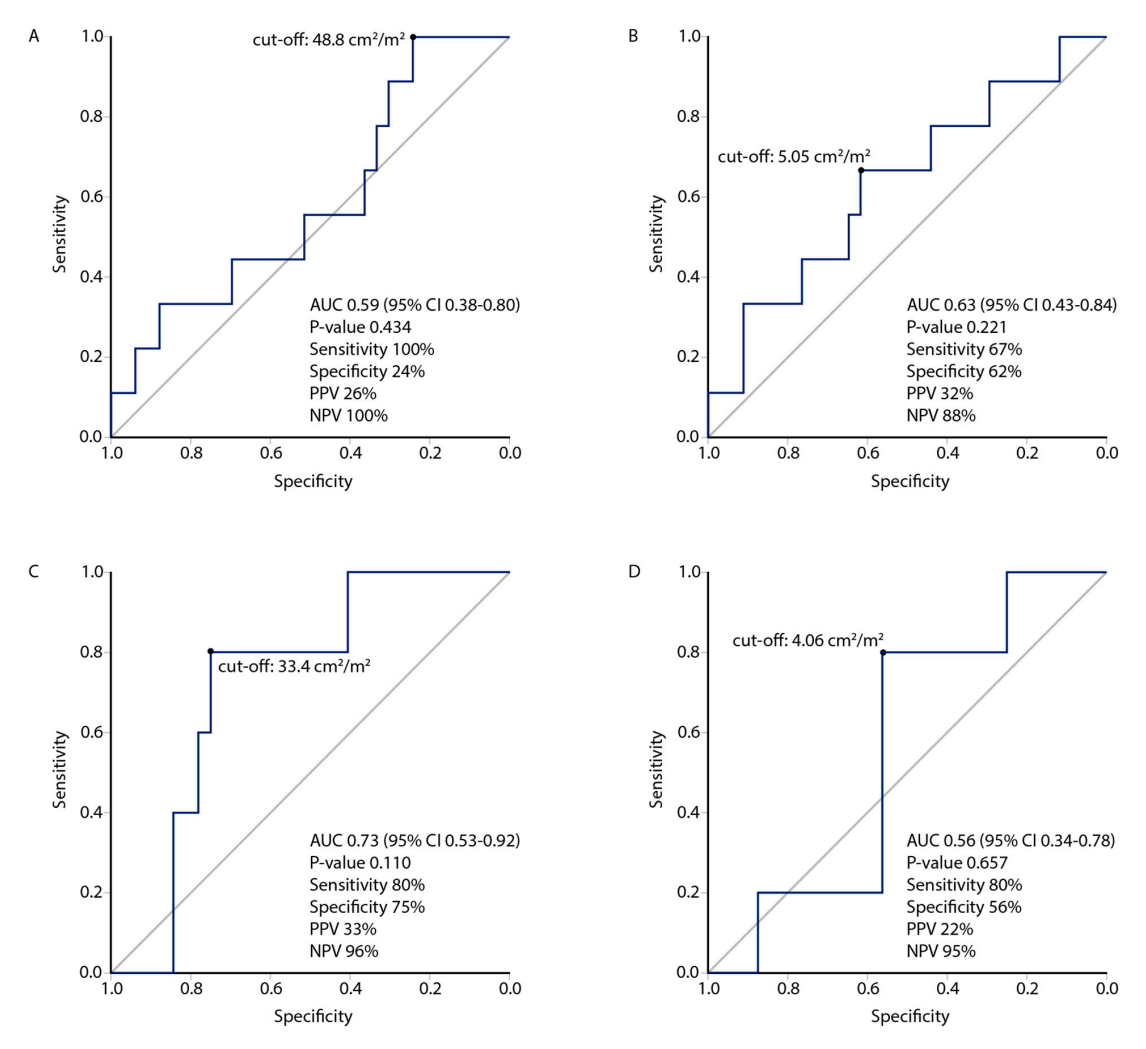
Optimized skeletal muscle index (SMI) and psoas muscle index (PMI) cut-offs to predict 3-month overall survival were determined by maximizing Youden’s indexes on the receiver operating characteristic curve. For men, the cut-offs were 48.8 cm^2^/m^2^ for SMI (**A**) and 5.05 cm^2^/m^2^ for PMI (**B**); for women 33.4 cm^2^/m^2^ for SMI (**C**) and 4.06 cm^2^/m^2^ for PMI (**D**).

ROC analyses were performed for SMI (n = 79) and PMI (n = 80) to predict 3-month OS rates (Figure 3). The optimized ROC cut-off values for SMI were 48.8 cm^2^/m^2^ and 33.4 cm^2^/m^2^, and for PMI 5.05 cm^2^/m^2^ and 4.06 cm^2^/m^2^ for men and women, respectively, as obtained by maximizing Youden’s indexes.

Median SMI cut-offs for men and women were 42.4 cm^2^/m^2^ and 35.0 cm^2^/m^2^, respectively; median PMI cut-offs were 5.06 cm^2^/m^2^ and 4.15 cm^2^/m^2^, respectively.

The optimized, median, and literature-referred SMI and PMI cut-offs and their univariate and multivariable associations with 3-month OS are presented in Table 2 [12,39,40,41,42,43,44] and demographic comparisons of the studies in Table S2.

### 3.4. Associations between Low Muscle Mass, Oncological Treatments, and Patient Characteristics

The associations between oncological treatments, clinical characteristics, and low muscle mass based on optimized SMI and PMI cut-offs are presented in Table 3 and Table 4, respectively, and further details are presented in Tables S3 and S4, respectively.

A low SMI did not associate with ECOG PS, FRAIL scale, clinical frailty scale, or other measures of functional status, whereas low PMI was associated with ECOG PS 3–4 (*p* = 0.047), clinical frailty scale 5–9 (*p* = 0.006), and an impaired hand-grip strength (*p* = 0.012).

Significant differences were noted in the measures of malnutrition. In the non-curative group, malnutrition according to GLIM was noted in 76% and 45% of patients with low and normal SMI, respectively (*p* = 0.027), and in the curative intent group according to the mini nutritional assessment-short form in 78% and 20%, respectively (*p* = 0.019). In the non-curative group, malnourishment according to GLIM was present in 82% and 52% of patients in the low and normal PMI groups, respectively (*p* = 0.029).

### 3.5. Three-Month Survival Analyses

**Figure 4:**
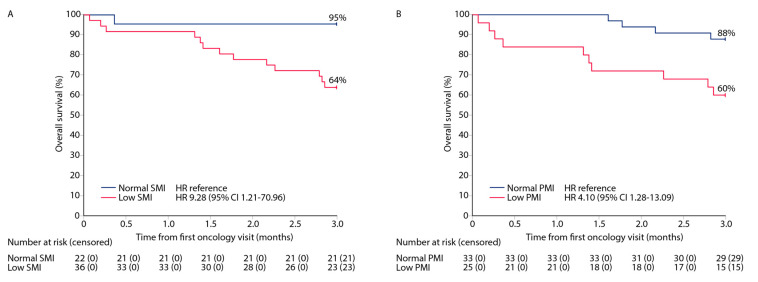
The Kaplan–Meier plots for 3-month OS rates in non-curative treatment intent patients based on optimized skeletal muscle index (SMI; (**A**)) and psoas muscle index (PMI; (**B**)) cut-offs determined with the Youden’s index method for patients in the non-curative intent treatment group.

The 3-month OS rates were 64% and 95% in the low and normal SMI groups, respectively, among the non-curatively treated patients with an HR of 9.28 (95% CI 1.2–71.0; Figure 4A). Respective 3-month OS rates were 60% and 88% in the low and normal PMI groups with an HR of 4.10 (95% CI 1.3–13.1; Figure 4B). Most deaths (13/14, 93%) occurred in the BSC group, with one death (due to stroke) out of 31 (3%) among oncologically treated patients. No patients in the curative treatment intent group died within 3 months.

### 3.6. Univariate and Multivariable Analyses for 3-Month Overall Survival in the Non-Curative Treatment Group

In the univariate analyses, low SMI, low PMI, and clinical frailty scale 5–9 were associated with impaired 3-month OS, whereas sex and ECOG PS 3–4 were not (Table 5).

The 3-month OS models included predefined predictors for cancer treatment outcome (low muscle mass, ECOG PS, and clinical frailty scale) and sex due to differences in muscle mass distributions. There were no significant interactions between SMI and ECOG PS (*p* = 0.954), SMI and clinical frailty scale (*p* = 0.929), or ECOG and clinical frailty scale (*p* = 0.947). Low SMI (HR 10.65 (95% CI 1.0–110)) remained statistically significant in the multivariable model (Table 5). None of the factors in the PMI model remained significant after the adjustment (Table 5).

The SMI [12,39,40,41,42] and PMI cut-offs [43,44] from the literature, in addition to our own median cut-offs, were included separately in the multivariable models that included low muscle mass, sex, ECOG PS, and clinical frailty scale. Of these, only the study median PMI cut-off was significant in the univariate analysis; however, none were independently associated with an impaired 3-month OS in multivariable analysis (Table 2).

### 3.7. Long-Term Overall Survival

The median reverse Kaplan–Meier follow-up was 29 months (IQR 24–38) with no patients lost to follow-up.

**Figure 5:**
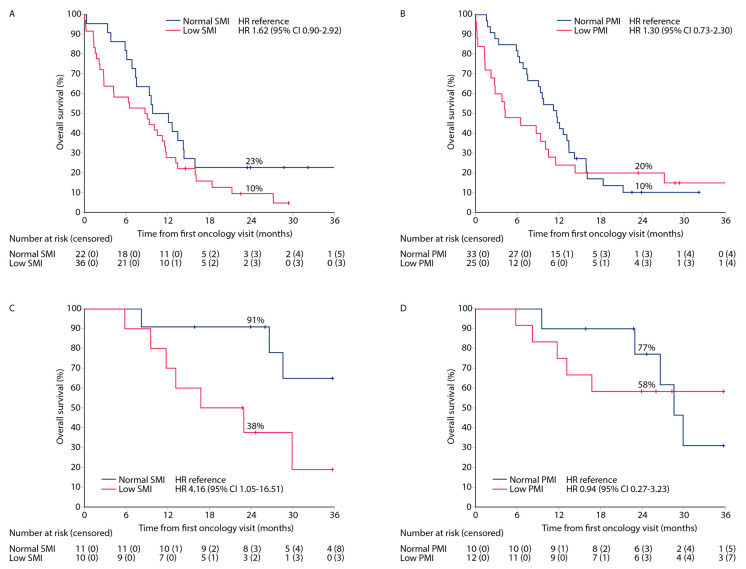
The Kaplan–Meier plots for long-term OS for patients in non-curative treatment intent group based on optimized skeletal muscle index (SMI; (**A**)) and psoas muscle index (PMI; (**B**)) and for patients in curative treatment intent group based on SMI (**C**) and PMI (**D**).

Patients in the non-curative group showed no statistically significant long-term OS associations between the low and normal SMI groups (HR 1.62 (95% CI 0.9–2.9)) with 24-month OS rates of 10% and 23%, respectively (Figure 5A). Low and normal PMI showed crossing of the curves after 12 months in the non-curative group (n = 58) (Figure 5B).

The 24-month OS rates in the palliative chemotherapy group (n = 31) were 23% and 31% in the low and normal SMI groups, respectively, with an HR of 1.19 (95% CI 0.53–2.7). Respective 24-month OS rates were 63% and 15% in the low and normal PMI groups.

Patients in the curative treatment group with low SMI had an impaired long-term survival (HR 4.16 (95% CI 1.1–17)) with 24-month OS rates of 38% and 91% in the low and normal SMI groups, respectively (Figure 5C), whereas PMI curves crossed after 24 months (Figure 5D).

## 4. Discussion

Our study suggests that low SMI is a predictor of worse 3-month OS independent of oncological and geriatric performance scores (ECOG PS and clinical frailty scale, respectively) in frail older adults with cancer. The study enrolled only patients at risk of frailty based on G8-screening (≤14/17 points) who underwent full CGA; fit older adults not at risk of frailty (>14/17 points) were excluded. Low PMI was predictive of 3-month survival in the univariate analysis but did not retain its statistical significance in the multivariable model. Low SMI was also associated with impaired long-term OS in the curative intent treatment group. Most patients allocated to BSC or follow-up (i.e., did not receive active oncological treatment) had low SMI. CT-determined low muscle mass could thus aid in the assessment of the treatability of these patients.

Several SMI and PMI cut-offs for low muscle mass in different patient populations have been proposed in the literature (Table 2). We noted that previously published SMI cut-offs by Prado et al. [12], Martin et al. [39], Baracos et al. [40], van Vledder et al. [41], and Camus et al. [42] classified most of our cohort’s older frail patients as having low muscle mass, and these classifications did not predict 3-month OS. In the studies by Prado et al. and Martin et al., the patients were significantly younger and had higher mean BMI values and notably higher SMI values than the patients in the current study. Patients in these two studies had a variety of primary tumors with stage IV disease in 38 and 52% of patients, respectively. The lower cut-off values by van Vledder et al. did not predict 3-month OS, probably due to the selection of fit patients undergoing liver resection for colorectal liver metastases, unlike our patient material, which excluded the fit. In the study by Camus et al., the patients’ ages were closer to the current cohort but with higher mean SMI values, probably reflecting the study population consisting of patients with lymphoma that mostly did not affect the gastrointestinal canal and thus their nutritional status. Similarly, the PMI cut-offs from surgical scenarios by Amini et al. [43] and Joglekar et al. [44] did not predict survival, but they classified patients into low and normal muscle mass groups more evenly than the cited SMI cut-offs from the literature. Indeed, PMI has been used mostly in surgical scenarios rather than oncological ones, and the cut-offs may not be applicable due to the differences in patient cohorts. In all the abovementioned studies, patients’ median ages were lower, and frailty was not reported (Table S2). Similar to this study, two of five studies assessing SMI included patients with a variety of solid tumors. Oncological therapy was assessed in five of the seven studies, as in our study, and surgical treatment in three studies. We clearly had the highest proportion of patients with locally advanced non-resectable, or metastatic tumors. To our knowledge, there are no frail older adult patient cohorts in the literature with which to compare our cut-offs. Larger cohorts are needed to validate our proposed cut-off values.

Our findings align with previous research showing that CT-based body composition analysis is a promising method for identifying patients with muscle-wasting conditions and impaired OS in many cancers [16,24,45,46,47,48,49,50]. Our results showed a non-significant association between low SMI and impaired long-term survival in the non-curative intent group. The association was statistically significant only in the curative intent treatment group that underwent surgical resection with or without neo-/adjuvant treatment. However, the baseline measurement of PMI seemed to lose its predictive value after 1 year in both instances.

BMI as a measure of body composition was not associated with OS or muscle mass in the current cohort. High BMI may be linked with worse cancer survival outcomes, as shown in two large meta-analyses [51,52], and in a large meta-analysis of early-stage breast cancer, obesity (BMI > 30) was found to be associated with a 75% and 34% mortality increase in premenopausal and postmenopausal women, respectively [53]. However, in a study of 41,015 patients with colorectal cancer, BMI values from 25 to 29 and from 30 to 35 were associated with better OS rates than normal BMI in adults ≥ 70 years of age (HRs of 0.77 (0.73–0.81) and 0.77 (0.69–0.87), respectively) with similar HRs for cancer-specific survival [54]. Similarly, in 471 adults ≥ 80 years of age undergoing a curative resection of stage I–III colorectal cancer, BMI ≥ 23 was found to be associated with better cancer-specific survival (0.54 (0.29–0.94) and OS (0.45 (0.30–0.65)) than BMI < 23 in multivariable analysis [55]. In addition, high BMI and better survival outcomes were reported in a meta-analysis of patients receiving immuno-oncological treatment [56]. The mechanism for the association between BMI and cancer mortality is unclear, but it has been suggested that a higher BMI may interfere with the effective delivery of oncological treatments [57] and contribute to the development of fatal comorbidities [58,59]. However, it is feasible that a higher BMI is reflective of higher energy and nutrient reserves [60] and is thus protective against cancer cachexia and mortality. The use of BMI alone to assess body composition in older adults is not advisable, considering the conflicting evidence, as it does not measure the highly variable proportions of lean or adipose tissue mass.

In line with our results, low muscle mass has previously been shown to be associated with a higher 3-month mortality in patients undergoing cancer surgery (e.g., for bladder cancer [61], abdominal emergencies in older adults [62], glioblastoma [63], liver metastases [64], and hepatocellular carcinoma [65]).

In the current study, only low PMI was associated with frailty as defined by clinical frailty scale points ranging between 5 and 9, ECOG PS 3 and 4, and impaired hand-grip strength. The psoas muscles are important in maintaining posture and are probably preserved in physically active patients (ECOG PS 0–2 and clinical frailty scale 0–4). It is unclear whether inactivity is reflected equally in reduced psoas or total muscle mass, but SMI is regarded as the gold standard surrogate for whole body muscle mass [20,24]. The finding regarding the SMI’s association with OS, but not with these measures of functional status, suggests that SMI probably reflects some aspects of fitness not readily detected at the geriatrician’s or oncologist’s appointment.

Low muscle mass with both muscle indexes was associated with impaired nutritional status, according to the GLIM classification. Many patients suffered from advanced diseases with inflammatory components (elevated CRP in 36% and hypoalbuminemia in 21%), which contribute to cachexia-related muscle loss (such as in pancreatic cancer). In addition, aging-related sarcopenia and frailty can cause malnutrition through several other mechanisms, including less activity, lower energy needs, food intake, and appetite, impaired cognition, and disrupted social functioning [66]. Previous surgery and fasting can also cause low muscle mass. The etiology of low muscle mass in this cohort remains unclear and is probably multifactorial.

The opportunistic use of CT-based body composition analysis is feasible and can complement geriatricians and oncologists in treatment decision-making. Our results suggest that low SMI is associated with treatment allocation to BSC, as 78% of these patients had low SMI. Based on our results, SMI would be preferred over PMI for the assessment of muscle mass in frail older adults, as only SMI remained significant in the multivariable 3-month OS model. We hypothesize that psoas measurements may be more susceptible to inter-reader and inter-study variability due to the smaller muscle area than the whole muscle area; hypothetically, small measurement errors could result in a comparatively large variability in PMI values.

The major limitation of this study is the small patient sample with varying types of cancer, stages, and treatment intents. There is thus a clear need for validation of the results in larger, homogenous patient cohorts. Another limitation (or strength) is the exclusion of fit older adults and the inclusion of only at-risk-of-frailty and CGA-assessed older adults. This affects long-term OS estimates, as these frail patients have a relatively short life expectancy, even without cancer [67,68]; patients in non-curative treatment have a less-than-12-month life expectancy due to a high unmet need in most cases, and the treatment intent necessitates the separation of the treatment intent groups. The exclusion of fit older adult patients (G8-screening > 14 points) suggests that our cut-off values are probably not applicable to these patients, warranting further research in this group. Furthermore, a time delay of 2 months between the CT scan and CGA was accepted, in which time the muscle mass may have changed, and we excluded newer scans taken after treatment initiation. Modest area-under-the-curve values, positive and negative predictive values, and broad CIs also indicate the need for further research in larger patient cohorts. The major strengths of the study are the primary endpoint and the patient population, where treatment decisions are extremely challenging. The 3-month OS, commonly used in oncological studies [64,69,70], was chosen to control the heterogeneity of the patient material and to serve as a surrogate for treatability decisions. The study is also a real-life application of body composition analysis, where all consecutive frail older adults were enrolled, and it reflects the current clinical practice of a modern geriatric oncological unit.

## 5. Conclusions

CT-determined low SMI is independently associated with impaired 3-month OS in frail older adults with cancer treated with non-curative intent. In addition, low PMI was associated with impaired functional status, and both muscle indexes were associated with malnutrition. Low SMI could thus be used as an indicator of treatability alongside oncologic and geriatric assessments and can help in the treatment decision between active oncological treatment and best supportive care or follow-up.

## Figures and Tables

**Table 1:** Patient characteristics for non-curative and curative treatment intent groups.

		All		Non-Curative		Curative		
		N = 80	%	n = 58	%	n = 22	%	OR (95% CI)
Age	75–80 years	39	49	28	48	11	50	1
	≥80 years	41	51	30	52	11	50	0.93 (0.4–2.5)
Sex	Female	37	46	28	48	9	41	1
	Male	43	54	30	52	13	59	1.35 (0.5–3.6)
Tumor site	Upper GI	37	46	30	52	7	32	1
	Lower GI	26	33	12	21	14	64	5.00 (1.6–15) *
	Other ^a^	17	21	16	28	1	5	0.27 (0.0–2.4)
Tumor stage	Local	13	16	0	0	13	59	1
	Metastatic or locally advanced	67	84	58	100	9	41	N/A
ECOG performance status	0 to 2	52	65	37	64	15	68	1
	3 to 4	22	28	15	26	7	32	1.15 (0.4–3.4)
	Not available	6	8	6	10	0	0	
Activities of daily living	Normal	52	65	35	60	17	77	1
	Impaired	24	30	19	33	5	23	0.54 (0.2–1.7)
	Not available	4	5	4	7	0	0	
Clinical frailty scale	1 to 4	48	60	35	60	13	59	1
	5 to 9	26	33	20	35	6	27	0.81 (0.3–2.5)
	Not available	6	8	3	5	3	14	
BMI	≥22 kg/m^2^	61	76	42	72	19	86	1
	<22 kg/m^2^	19	24	16	28	3	14	0.41 (0.1–1.6)
GLIM ^b^	Normal	31	39	19	33	12	55	1
	Malnourishment	44	55	34	59	10	46	0.47 (0.2–1.39)
	Not available	5	6	5	9	0	0	
Hand-grip strength test	Normal	48	60	36	62	12	55	1
	Impaired	22	28	13	22	9	41	2.08 (0.7–6.1)
	Not available	10	13	9	16	1	5	
Albumin	≥30 g/L	41	51	28	48	13	59	1
	<30 g/L	17	21	14	24	3	14	0.46 (0.1–1.9)
	Not available	22	28	16	28	6	27	
SMI ^c^	Normal	33	42	22	38	11	52	1
	Low	46	58	36	62	10	48	0.56 (0.2–1.5)
PMI ^c^	Normal	43	54	33	57	10	46	1
	Low	37	46	25	43	12	55	1.58 (0.6–4.3)
Treatment decision	Oncological treatments	41	51	31	53	10	46	1
	Follow-up or BSC ^d^	39	49	27	47	12	55	1.38 (0.5–3.7)
Survival at 3 months	Alive	66	83	44	76	22	100	1
	Deceased	14	18	14	24	0	0	N/A

^a^ Other cancers included 7 genitourinary cancers, 4 breast cancers, 4 lung cancers, 1 sarcoma of the lower limb, and 1 thymus carcinoma; ^b^ Global Leadership Initiative on Malnutrition classification; ^c^ patients were divided into normal and low SMI/PMI categories using our cut-offs obtained by maximizing the Youden’s index on the receiver operator characteristic curve; ^d^ best supportive care; * indicates statistical significance (*p* ≤ 0.05).

**Table 2:** Univariate and multivariable Cox regression analyses between different muscle index cut-offs and 3-month OS in the non-curative intent group.

		All Patients	Non-Curative Patients	Univariate	Multivariable ^a^
	Cut-Off	N	n Univariate/n Multivariable	HR 95% CI	HR 95% CI
Study SMI (Youden) ^b^	Normal	33	22/22	1	1
	Low	46	36/29	9.28 (1.2–71) *	10.65 (1.0–110) *
Study SMI (median) ^c^	Normal	40	29/28	1	1
	Low	39	29/23	2.06 (0.7–6.2)	2.21 (0.6–8.4)
Prado et al. SMI ^d^ [12]	Normal	10	7/7	1	1
	Low	69	51/44	25.21 (0.0–19,000)	N/A
Martin et al. SMI ^e^ [39]	Normal	13	9/8	1	1
	Low	66	49/43	1.19 (0.3–5.3)	1.00 (0.1–8.8)
Baracos et al. SMI ^f^ [40]	Normal	10	7/7	1	1
	Low	69	51/44	25.21 (0.0–19,400)	N/A
van Vledder et al. SMI ^g^ [41]	Normal	21	14/13	1	1
	Low	58	44/38	0.86 (0.3–2.7)	1.01 (0.3–4.5)
Camus et al. SMI ^h^ [42]	Normal	9	7/7	1	1
	Low	70	51/44	25.21 (0.3–19,000)	N/A
Study PMI (Youden) ^i^	Normal	43	33/32	1	1
	Low	37	25/18	4.10 (1.3–13) *	2.23 (0.6–8.9)
Study PMI (median) ^j^	Normal	41	31/30	1	1
	Low	39	27/21	4.48 (1.1–11) *	1.56 (0.4–6.5)
Amini et al. PMI ^k^ [43]	Normal	34	26/26	1	1
	Low	46	32/25	3.46 (1.0–12)	1.84 (0.4–7.7)
Joglekar et al. PMI ^l^ [44]	Normal	37	28/28	1	1
	Low	43	30/23	2.74 (0.9–8.7)	1.31 (0.3–5.2)

^a^ Adjusted for sex (female vs. male), ECOG performance status (0–2 vs. 3–4), clinical frailty scale (1–4 vs. 5–9); ^b^ cut-offs for SMI by Youden method were 48.8 cm^2^/m^2^ for men and 33.4 cm^2^/m^2^ for women; ^c^ median cut-offs for SMI were 42.4 cm^2^/m^2^ for men and 35.0 cm^2^/m^2^ for women; ^d^ cut-offs for SMI by Prado et al. [12], 52.4 cm^2^/m^2^ for men and 38.5 cm^2^/m^2^ for women; ^e^ cut-offs for SMI by Martin et al. [39] 43 cm^2^/m^2^ for men with BMI < 25 kg/m^2^, 53 cm^2^/m^2^ for men with BMI ≥ 25 kg/m^2^, and 41 cm^2^/m^2^ for women regardless of BMI; ^f^ cut-offs for SMI by Baracos et al. [40], 55.4 cm^2^/m^2^ for men and 38.9 cm^2^/m^2^ for women; ^g^ cut-offs for SMI by van Vledder et al. [41], 43.8 cm^2^/m^2^ for men and 38.9 cm^2^/m^2^ for women; ^h^ cut-offs for SMI by Camus et al. [42], 55.8 cm^2^/m^2^ for men and 38.9 cm^2^/m^2^ for women; ^i^ cut-offs for PMI by Youden method were 5.05 cm^2^/m^2^ for men and 4.06 cm^2^/m^2^ for women; ^j^ median cut-offs for PMI were 5.06 cm^2^/m^2^ for men and 4.15 cm^2^/m^2^ for women; ^k^ cut-offs for PMI by Amini et al. [43], 5.642 cm^2^/m^2^ for men and 4.145 cm^2^/m^2^ for women; ^l^ cut-offs for PMI by Joglekar et al. [44], 5.2 cm^2^/m^2^ for men and 4.0 cm^2^/m^2^ for women; N/A = not applicable; * indicates statistical significance (*p* ≤ 0.05).

**Table 3:** Associations between optimized low skeletal muscle index (SMI) and patient characteristics.

		Non-Curative Treatment			Curative Treatment		
		Normal SMI	Low SMI ^a^		Normal SMI	Low SMI ^a^	
		n = 22	n = 36	OR (95% CI)	n = 11	n = 10	OR (95% CI)
		n (%)	n (%)		n (%)	n (%)	
Age	75–80 years	8 (29)	20 (71)	1	8 (80)	2 (20)	1
	≥80 years	14 (47)	16 (53)	0.46 (0.2–1.4)	3 (27)	8 (73)	10.67 (1.4–82) *
Sex	Female	19 (68)	9 (32)	1	6 (67)	3 (33)	1
	Male	3 (10)	27 (90)	19.00 (4.5–80) *	5 (42)	7 (58)	2.80 (0.5–17)
Tumor site	Upper GI	8 (27)	22 (73)	1	4 (57)	3 (43)	1
	Lower GI	4 (33)	8 (67)	0.73 (0.2–3.1)	7 (54)	6 (46)	1.14 (0.2–7.3)
	Other ^b^	10 (63)	6 (38)	0.22 (0.1–0.8)	0 (0)	1 (100)	N/A
Tumor stage	Local	0 (0)	0 (0)	1	7 (54)	6 (46)	1
	Metastatic or locally advanced	22 (38)	36 (62)	N/A	4 (50)	4 (50)	1.17 (0.2–6.8)
ECOG performance status	0 to 2	16 (43)	21 (57)	1	9 (60)	6 (40)	1
	3 to 4	6 (40)	9 (60)	1.14 (0.3–3.9)	2 (33)	4 (67)	3.00 (0.4–22)
	Not available	0 (0)	6 (100)		0 (0)	0 (0)	
Activities of daily living	Normal	14 (40)	21 (60)	1	8 (50)	8 (50)	1
	Impaired	7 (37)	12 (63)	1.14 (0.4–3.6)	3 (60)	2 (40)	0.67 (0.1–5.1)
	Not available	1 (25)	3 (75)		0 (0)	0 (0)	
Clinical frailty scale	1 to 4	15 (43)	20 (57)	1	8 (62)	5 (39)	1
	5 to 9	7 (35)	13 (65)	1.39 (0.4–4.3)	2 (40)	3 (60)	2.40 (0.3–20)
	Not available	0 (0)	3 (100)		1 (33)	2 (67)	
BMI	≥22 kg/m^2^	17 (41)	25 (60)	1	10 (56)	8 (44)	1
	<22 kg/m^2^	5 (31)	11 (69)	1.50 (0.4–5.1)	1 (33)	2 (67)	2.50 (0.2–33)
GLIM ^c^	Normal	11 (58)	8 (42)	1	6 (55)	5 (46)	1
	Malnourishment	9 (27)	25 (74)	3.82 (1.2–13) *	5 (50)	5 (50)	1.20 (0.2–6.7)
	Not available	2 (40)	3 (60)		0 (0)	0 (0)	
Hand-grip strength test	Normal	16 (44)	20 (56)	1	6 (50)	6 (50)	1
	Impaired	3 (23)	10 (77)	2.67 (0.6–11)	4 (50)	4 (50)	1.00 (0.2–6.0)
	Not available	3 (33)	6 (67)		1 (100)	0 (0)	
Albumin	≥30 g/L	12 (43)	16 (57)	1	8 (67)	4 (33)	1
	<30 g/L	2 (14)	12 (86)	4.50 (0.8–24)	1 (33)	2 (67)	4.00 (0.3–59)
	Not available	8 (50)	8 (50)		2 (33)	4 (67)	
Treatment decision	Oncological treatment	16 (52)	15 (48)	1	6 (60)	4 (40)	1
	Follow-up or BSC ^d^	6 (22)	21 (78)	3.73 (1.2–12) *	5 (46)	6 (55)	1.80 (0.3–10)
Survival at 3 months	Alive	21 (48)	23 (52)	1	11 (52)	10 (48)	1
	Deceased	1 (7)	13 (93)	11.87 (1.4–99) *	0 (0)	0 (0)	N/A

^a^ Patients with normal and low SMI according to our cut-offs obtained by maximizing the Youden’s index on the receiver operator characteristic curve. ^b^ Other cancers included 7 genitourinary cancers, 4 breast cancers, 4 lung cancers, 1 sarcoma of the lower limb, and 1 thymus carcinoma. ^c^ Global Leadership Initiative on Malnutrition classification. ^d^ Best supportive care. * indicates statistical significance (*p* ≤ 0.05).

**Table 4:** Associations between optimized low psoas muscle index (PMI) and patient characteristics.

		Non-Curative Treatment			Curative Treatment		
		Normal PMI	Low PMI ^a^		Normal PMI	Low PMI ^a^	
		n = 33	n = 25	OR (95% CI)	n = 10	n = 12	OR (95% CI)
		n (%)	n (%)		n (%)	n (%)	
Age	75–80 years	16 (57)	12 (43)	1	5 (46)	6 (55)	1
	≥80 years	17 (57)	13 (43)	1.02 (0.4–2.9)	5 (46)	6 (55)	1.00 (0.2–5.4)
Sex	Female	16 (57)	12 (43)	1	3 (33)	6 (67)	1
	Male	17 (57)	13 (43)	1.02 (0.4–2.9)	7 (54)	6 (46)	0.43 (0.1–2.5)
Tumor site	Upper GI	18 (60)	12 (40)	1	1 (14)	6 (86)	1
	Lower GI	5 (42)	7 (58)	2.10 (0.5–8.2)	8 (57)	6 (43)	0.13 (0.0–1.3)
	Other ^b^	10 (63)	6 (38)	0.9 (0.3–3.1)	1 (100)	0 (0)	N/A
Tumor stage	Local	0 (0)	0 (0)	1	7 (54)	6 (46)	1
	Metastatic or locally advanced	33 (57)	25 (43)	N/A	3 (33)	6 (67)	2.33 (0.4–14)
ECOG performance status	0 to 2	26 (70)	11 (30)	1	8(53)	7 (47)	1
	3 to 4	6 (40)	9 (60)	3.55 (1.0–12) *	2 (29)	5 (71)	2.86 (0.4–20)
	Not available	1 (17)	5 (83)		0 (0)	0 (0)	
Activities of daily living	Normal	22 (63)	13 (37)	1	9 (53)	8 (47)	1
	Impaired	10 (53)	9 (47)	1.52 (0.5–4.7)	1 (20)	4 (80)	4.50 (0.4–49)
	Not available	1 (25)	3 (75)		0 (0)	0 (0)	
Clinical frailty scale	1 to 4	26 (74)	9 (26)	1	6 (46)	7 (54)	1
	5 to 9	7 (35)	13 (65)	5.37 (1.6–18) *	1 (17)	5 (83)	4.29 (0.4–48)
	Not available	0 (0)	3 (100)		3 (100)	0 (0)	
BMI	≥22 kg/m^2^	25 (60)	17 (41)	1	10 (53)	9 (47)	1
	<22 kg/m^2^	8 (50)	8 (50)	1.47 (0.5–4.7)	0 (0)	3 (100)	N/A
GLIM ^c^	Normal	15 (79)	4 (21)	1	6 (50)	6 (50)	1
	Malnourishment	16 (47)	18 (53)	4.22 (1.2–15) *	4 (40)	6 (60)	1.50 (0.3–8.2)
	Not available	2 (40)	3 (60)		0 (0)	0 (0)	
Hand-grip strength	Normal	26 (72)	10 (28)	1	4 (33)	8 (67)	1
	Impaired	4 (31)	9 (69)	5.85 (1.5–23) *	5 (56)	4 (44)	0.40 (0.1–2.4)
	Not available	3 (33)	6 (67)		1 (100)	0 (0)	
Albumin	≥30	19 (68)	9 (32)	1	7 (54)	6 (46)	1
	<30	6 (43)	8 (57)	2.82 (0.8–11)	0 (0)	3 (100)	N/A
	Not available	8 (50)	8 (50)		3 (50)	3 (50)	
Treatment decision	Oncological treatment	23 (74)	8 (26)	1	3 (30)	7 (70)	1
	Follow-up or BSC ^d^	10 (37)	17 (63)	4.89 (1.6–15) *	7 (58)	5 (42)	0.31 (0.1–1.8)
Survival at 3 months	Alive	29 (66)	15 (34)	1	10 (46)	12 (55)	1
	Deceased	4 (29)	10 (71)	4.83 (1.3–18) *	0 (0)	0 (0)	N/A

^a^ Patients with normal and low PMI categories according to our cut-offs obtained by maximizing the Youden’s index on the receiver operator characteristic curve. ^b^ Other cancers included 7 genitourinary cancers, 4 breast cancers, 4 lung cancers, 1 sarcoma of the lower limb, and 1 thymus carcinoma. ^c^ Global Leadership Initiative on Malnutrition classification. ^d^ Best supportive care. * indicates statistical significance (*p* ≤ 0.05).

**Table 5:** Univariate and multivariable Cox regression analyses between 3-month OS and optimized muscle indexes (SMI/PMI), sex, ECOG performance status and clinical frailty scale in the non-curative intent treatment group.

		SMI		PMI	
	Patient n	Univariate	Multivariable	Univariate	Multivariable
	Univariate/Multivariable	HR (95% CI)	HR (95% CI)	HR (95% CI)	HR (95% CI)
Muscle mass					
Normal	SMI 22/22; PMI 33/32	1	1	1	1
Low ^a^	SMI 36/29; PMI 25/18	9.28 (1.2–71) *	10.65 (1.0–110) *	4.10 (1.3–13) *	2.23 (0.6–8.9)
Sex					
Women	28/25	1	1	1	1
Men	30/26	1.68 (0.6–5.0)	0.57 (0.1–2.8)	1.68 (0.6–5.0)	2.1 (0.5–8.1)
ECOG PS					
0 to 2	37/36	1	1	1	1
3 to 4	15/15	3.01 (0.9–10)	2.34 (0.6–9.8)	3.01 (0.9–10)	1.80 (0.4–7.2)
Clinical frailty scale					
1 to 4	35/33	1	1	1	1
5 to 9	20/18	3.35 (1.1–10) *	2.11 (0.5–8.6)	3.35 (1.1–10) *	1.66 (0.4–7.6)

^a^ Patients were divided into normal and low SMI/PMI categories using our SMI/PMI cut-offs obtained by maximizing Youden’s indexes on the receiver operating characteristic curve; * indicates statistical significance (*p* ≤ 0.05).

## Data Availability

All the relevant data are presented in the article.

## Supplementary Materials

The following supporting information can be downloaded at: https://www.mdpi.com/article/10.3390/cancers15133398/s1, Table S1: Extended patient characteristics for non-curative and curative treatment intent groups; Table S2: Extended associations between optimized low skeletal muscle index (SMI) and patient characteristics; Table S3: Extended associations between optimized low psoas muscle index (PMI) and patient characteristics; Table S4: Demographic comparisons of the referred SMI/PMI cut-off studies in the literature.

## References

[1] Balducci L., Aapro M. (2005). Epidemiology of Cancer and Aging. Biol. Basis Geriatr. Oncol..

[2] Handforth C., Clegg A., Young C., Simpkins S., Seymour M.T., Selby P.J., Young J. (2015). The Prevalence and Outcomes of Frailty in Older Cancer Patients: A Systematic Review. Ann. Oncol..

[3] Huynh L., Moore J. (2021). Palliative and End-of-Life Care for the Older Adult with Cancer. Curr. Opin. Support. Palliat. Care.

[4] Veasey Rodrigues H., Baracos V.E., Wheler J.J., Parsons H.A., Hong D.S., Naing A., Fu S., Falchoock G., Tsimberidou A.M., Piha-Paul S. (2013). Body Composition and Survival in the Early Clinical Trials Setting. Eur. J. Cancer.

[5] Extermann M., Aapro M., Topinkova E., Bernabei R., Cohen H.J., Droz J.-P., Lichtman S., Mor V., Monfardini S., Repetto L. (2005). Use of Comprehensive Geriatric Assessment in Older Cancer Patients: Recommendations from the Task Force on CGA of the International Society of Geriatric Oncology (SIOG). Crit. Rev. Oncol. Hematol..

[6] Mohile S.G., Mohamed M.R., Culakova E., Xu H., Loh K.P., Magnuson A., Flannery M.A., Ramsdale E.E., Dunne R.F., Gilmore N. (2020). A Geriatric Assessment (GA) Intervention to Reduce Treatment Toxicity in Older Patients with Advanced Cancer: A University of Rochester Cancer Center NCI Community Oncology Research Program Cluster Randomized Clinical Trial (CRCT). J. Clin. Oncol..

[7] Li D., Sun C.-L., Kim H., Soto-Perez-de-Celis E., Chung V., Koczywas M., Fakih M., Chao J., Cabrera Chien L., Charles K. (2021). Geriatric Assessment-Driven Intervention (GAIN) on Chemotherapy-Related Toxic Effects in Older Adults with Cancer: A Randomized Clinical Trial. JAMA Oncol..

[8] Soo W.-K., King M., Pope A., Parente P., Darzins P., Davis I.D. (2020). Integrated Geriatric Assessment and Treatment (INTEGERATE) in Older People with Cancer Planned for Systemic Anticancer Therapy. J. Clin. Oncol..

[9] Chuang M.-H., Chen J.-Y., Tsai W.-W., Lee C.-W., Lee M.-C., Tseng W.-H., Hung K.-C. (2022). Impact of Comprehensive Geriatric Assessment on the Risk of Adverse Events in the Older Patients Receiving Anti-Cancer Therapy: A Systematic Review and Meta-Analysis. Age Ageing.

[10] Cruz-Jentoft A.J., Bahat G., Bauer J., Boirie Y., Bruyère O., Cederholm T., Cooper C., Landi F., Rolland Y., Sayer A.A. (2019). Sarcopenia: Revised European Consensus on Definition and Diagnosis. Age Ageing.

[11] Arends J., Strasser F., Gonella S., Solheim T.S., Madeddu C., Ravasco P., Buonaccorso L., de van der Schueren M.A.E., Baldwin C., Chasen M. (2021). Cancer Cachexia in Adult Patients: ESMO Clinical Practice Guidelines. ESMO Open.

[12] Prado C.M.M., Lieffers J.R., McCargar L.J., Reiman T., Sawyer M.B., Martin L., Baracos V.E. (2008). Prevalence and Clinical Implications of Sarcopenic Obesity in Patients with Solid Tumours of the Respiratory and Gastrointestinal Tracts: A Population-Based Study. Lancet Oncol..

[13] Sugiyama K., Narita Y., Mitani S., Honda K., Masuishi T., Taniguchi H., Kadowaki S., Ura T., Ando M., Tajika M. (2018). Baseline Sarcopenia and Skeletal Muscle Loss During Chemotherapy Affect Survival Outcomes in Metastatic Gastric Cancer. Anticancer Res..

[14] van Rijn-Dekker M.I., van den Bosch L., van den Hoek J.G.M., Bijl H.P., van Aken E.S.M., van der Hoorn A., Oosting S.F., Halmos G.B., Witjes M.J.H., van der Laan H.P. (2020). Impact of Sarcopenia on Survival and Late Toxicity in Head and Neck Cancer Patients Treated with Radiotherapy. Radiother. Oncol..

[15] Shachar S.S., Deal A.M., Weinberg M., Nyrop K.A., Williams G.R., Nishijima T.F., Benbow J.M., Muss H.B. (2017). Skeletal Muscle Measures as Predictors of Toxicity, Hospitalization, and Survival in Patients with Metastatic Breast Cancer Receiving Taxane-Based Chemotherapy. Clin. Cancer Res..

[16] Cushen S.J., Power D.G., Murphy K.P., McDermott R., Griffin B.T., Lim M., Daly L., MacEneaney P., O’ Sullivan K., Prado C.M. (2016). Impact of Body Composition Parameters on Clinical Outcomes in Patients with Metastatic Castrate-Resistant Prostate Cancer Treated with Docetaxel. Clin. Nutr. ESPEN.

[17] Daly L.E., Ní Bhuachalla É.B., Power D.G., Cushen S.J., James K., Ryan A.M. (2018). Loss of Skeletal Muscle during Systemic Chemotherapy Is Prognostic of Poor Survival in Patients with Foregut Cancer. J. Cachexia Sarcopenia Muscle.

[18] Han J.S., Ryu H., Park I.J., Kim K.W., Shin Y., Kim S.O., Lim S.-B., Kim C.W., Yoon Y.S., Lee J.L. (2020). Association of Body Composition with Long-Term Survival in Non-Metastatic Rectal Cancer Patients. Cancer Res. Treat..

[19] Portal D., Hofstetter L., Eshed I., Dan-Lantsman C., Sella T., Urban D., Onn A., Bar J., Segal G. (2019). L3 Skeletal Muscle Index (L3SMI) Is a Surrogate Marker of Sarcopenia and Frailty in Non-Small Cell Lung Cancer Patients. Cancer Manag. Res..

[20] Couderc A.-L., Liuu E., Boudou-Rouquette P., Poisson J., Frelaut M., Montégut C., Mebarki S., Geiss R., Ap Thomas Z., Noret A. (2023). Pre-Therapeutic Sarcopenia among Cancer Patients: An Up-to-Date Meta-Analysis of Prevalence and Predictive Value during Cancer Treatment. Nutrients.

[21] Wendrich A.W., Swartz J.E., Bril S.I., Wegner I., de Graeff A., Smid E.J., de Bree R., Pothen A.J. (2017). Low Skeletal Muscle Mass Is a Predictive Factor for Chemotherapy Dose-Limiting Toxicity in Patients with Locally Advanced Head and Neck Cancer. Oral Oncol..

[22] Tan B.H.L., Brammer K., Randhawa N., Welch N.T., Parsons S.L., James E.J., Catton J.A. (2015). Sarcopenia Is Associated with Toxicity in Patients Undergoing Neo-Adjuvant Chemotherapy for Oesophago-Gastric Cancer. Eur. J. Surg. Oncol..

[23] Nipp R.D., Fuchs G., El-Jawahri A., Mario J., Troschel F.M., Greer J.A., Gallagher E.R., Jackson V.A., Kambadakone A., Hong T.S. (2018). Sarcopenia Is Associated with Quality of Life and Depression in Patients with Advanced Cancer. Oncologist.

[24] Tolonen A., Pakarinen T., Sassi A., Kyttä J., Cancino W., Rinta-Kiikka I., Pertuz S., Arponen O. (2021). Methodology, Clinical Applications, and Future Directions of Body Composition Analysis Using Computed Tomography (CT) Images: A Review. Eur. J. Radiol..

[25] Soubeyran P., Bellera C., Goyard J., Heitz D., Curé H., Rousselot H., Albrand G., Servent V., Jean O.S., van Praagh I. (2014). Screening for Vulnerability in Older Cancer Patients: The ONCODAGE Prospective Multicenter Cohort Study. PLoS ONE.

[26] Osterlund P., Salminen T., Soveri L.-M., Kallio R., Kellokumpu I., Lamminmäki A., Halonen P., Ristamäki R., Lantto E., Uutela A. (2021). Repeated Centralized Multidisciplinary Team Assessment of Resectability, Clinical Behavior, and Outcomes in 1086 Finnish Metastatic Colorectal Cancer Patients (RAXO): A Nationwide Prospective Intervention Study. Lancet Reg. Health Eur..

[27] Eisenhauer E.A., Therasse P., Bogaerts J., Schwartz L.H., Sargent D., Ford R., Dancey J., Arbuck S., Gwyther S., Mooney M. (2009). New Response Evaluation Criteria in Solid Tumours: Revised RECIST Guideline (Version 1.1). Eur. J. Cancer.

[28] 3D Slicer. https://www.slicer.org/.

[29] Peng Y.-C., Wu C.-H., Tien Y.-W., Lu T.-P., Wang Y.-H., Chen B.-B. (2021). Preoperative Sarcopenia Is Associated with Poor Overall Survival in Pancreatic Cancer Patients Following Pancreaticoduodenectomy. Eur. Radiol..

[30] Oken M.M., Creech R.H., Tormey D.C., Horton J., Davis T.E., McFadden E.T., Carbone P.P. (1982). Toxicity and Response Criteria of the Eastern Cooperative Oncology Group. Am. J. Clin. Oncol..

[31] van Kan G.A., Rolland Y.M., Morley J.E., Vellas B. (2008). Frailty: Toward a Clinical Definition. J. Am. Med. Dir. Assoc..

[32] Rockwood K., Song X., MacKnight C., Bergman H., Hogan D.B., McDowell I., Mitnitski A. (2005). A Global Clinical Measure of Fitness and Frailty in Elderly People. Can. Med. Assoc. J..

[33] Finlayson M., Mallinson T., Barbosa V.M. (2005). Activities of Daily Living (ADL) and Instrumental Activities of Daily Living (IADL) Items Were Stable over Time in a Longitudinal Study on Aging. J. Clin. Epidemiol..

[34] Aromaa A., Koskinen S. (2004). Health and Functional Capacity in Finland. Baseline Results of the Health 2000 Health Examination Survey. National Public Health Institute. https://urn.fi/URN:NBN:fi-fe201204193452.

[35] Rubenstein L.Z., Harker J.O., Salvà A., Guigoz Y., Vellas B. (2001). Screening for Undernutrition in Geriatric Practice: Developing the Short-Form Mini-Nutritional Assessment (MNA-SF). J. Gerontol. A Biol. Sci. Med. Sci..

[36] Cederholm T., Jensen G.L., Correia M.I.T.D., Gonzalez M.C., Fukushima R., Higashiguchi T., Baptista G., Barazzoni R., Blaauw R., Coats A. (2019). GLIM Criteria for the Diagnosis of Malnutrition—A Consensus Report from the Global Clinical Nutrition Community. Clin. Nutr..

[37] Proctor M.J., Morrison D.S., Talwar D., Balmer S.M., O’Rreilly D., Foulis A.K., Horgan P.G., McMillan D.C. (2011). An Inflammation-Based Prognostic Score (MGPS) Predicts Cancer Survival Independent of Tumour Site: A Glasgow Inflammation Outcome Study. Br. J. Cancer.

[38] Sorbye H., Köhne C.-H., Sargent D.J., Glimelius B. (2007). Patient Characteristics and Stratification in Medical Treatment Studies for Metastatic Colorectal Cancer: A Proposal for Standardization of Patient Characteristic Reporting and Stratification. Ann. Oncol..

[39] Martin L., Birdsell L., Macdonald N., Reiman T., Clandinin M.T., McCargar L.J., Murphy R., Ghosh S., Sawyer M.B., Baracos V.E. (2013). Cancer Cachexia in the Age of Obesity: Skeletal Muscle Depletion Is a Powerful Prognostic Factor, Independent of Body Mass Index. J. Clin. Oncol..

[40] Baracos V.E., Rreiman T., Mourtzakis M., Gioulbasanis I., Antoun S. (2010). Body Composition in Patients with Non-Small Cell Lung Cancer: A Contemporary View of Cancer Cachexia with the Use of Computed Tomography Image Analysis. Am. J. Clin. Nutr..

[41] van Vledder M.G., Levolger S., Ayez N., Verhoef C., Tran T.C.K., IJzermans J.N.M. (2012). Body Composition and Outcome in Patients Undergoing Resection of Colorectal Liver Metastases. Br. J. Surg..

[42] Camus V., Lanic H., Kraut J., Modzelewski R., Clatot F., Picquenot J.M., Contentin N., Lenain P., Groza L., Lemasle E. (2014). Prognostic Impact of Fat Tissue Loss and Cachexia Assessed by Computed Tomography Scan in Elderly Patients with Diffuse Large B-Cell Lymphoma Treated with Immunochemotherapy. Eur. J. Haematol..

[43] Amini N., Spolverato G., Gupta R., Margonis G.A., Kim Y., Wagner D., Rezaee N., Weiss M.J., Wolfgang C.L., Makary M.M. (2015). Impact Total Psoas Volume on Short- and Long-Term Outcomes in Patients Undergoing Curative Resection for Pancreatic Adenocarcinoma: A New Tool to Assess Sarcopenia. J. Gastrointest. Surg..

[44] Joglekar S., Asghar A., Mott S.L., Johnson B.E., Button A.M., Clark E., Mezhir J.J. (2015). Sarcopenia Is an Independent Predictor of Complications Following Pancreatectomy for Adenocarcinoma. J. Surg. Oncol..

[45] Deluche E., Leobon S., Desport J.C., Venat-Bouvet L., Usseglio J., Tubiana-Mathieu N. (2018). Impact of Body Composition on Outcome in Patients with Early Breast Cancer. Support Care Cancer.

[46] Su H., Ruan J., Chen T., Lin E., Shi L. (2019). CT-Assessed Sarcopenia Is a Predictive Factor for Both Long-Term and Short-Term Outcomes in Gastrointestinal Oncology Patients: A Systematic Review and Meta-Analysis. Cancer Imaging.

[47] Shirdel M., Andersson F., Myte R., Axelsson J., Rutegård M., Blomqvist L., Riklund K., van Guelpen B., Palmqvist R., Gylling B. (2020). Body Composition Measured by Computed Tomography Is Associated with Colorectal Cancer Survival, also in Early-Stage Disease. Acta Oncol..

[48] Basile D., Parnofiello A., Vitale M.G., Cortiula F., Gerratana L., Fanotto V., Lisanti C., Pelizzari G., Ongaro E., Bartoletti M. (2019). The IMPACT Study: Early Loss of Skeletal Muscle Mass in Advanced Pancreatic Cancer Patients. J. Cachexia Sarcopenia Muscle.

[49] Fukushima H., Nakanishi Y., Kataoka M., Tobisu K., Koga F. (2016). Prognostic Significance of Sarcopenia in Patients with Metastatic Renal Cell Carcinoma. J. Urol..

[50] Jung A.R., Roh J.-L., Kim J.S., Kim S.-B., Choi S.-H., Nam S.Y., Kim S.Y. (2019). Prognostic Value of Body Composition on Recurrence and Survival of Advanced-Stage Head and Neck Cancer. Eur. J. Cancer.

[51] Jiralerspong S., Kim E.S., Dong W., Feng L., Hortobagyi G.N., Giordano S.H. (2013). Obesity, Diabetes, and Survival Outcomes in a Large Cohort of Early-Stage Breast Cancer Patients. Ann. Oncol..

[52] Petrelli F., Cortellini A., Indini A., Tomasello G., Ghidini M., Nigro O., Salati M., Dottorini L., Iaculli A., Varricchio A. (2021). Association of Obesity with Survival Outcomes in Patients with Cancer: A Systematic Review and Meta-Analysis. JAMA Netw. Open.

[53] Chan D.S.M., Vieira A.R., Aune D., Bandera E.V., Greenwood D.C., McTiernan A., Navarro Rosenblatt D., Thune I., Vieira R., Norat T. (2014). Body Mass Index and Survival in Women with Breast Cancer-Systematic Literature Review and Meta-Analysis of 82 Follow-up Studies. Ann. Oncol..

[54] Chiu C.-C., Ho C.-H., Hung C.-M., Chao C.-M., Lai C.-C., Chen C.-M., Liao K.-M., Wang J.-J., Wu Y.-C., Shi H.-Y. (2021). Correlation of Body Mass Index with Oncologic Outcomes in Colorectal Cancer Patients: A Large Population-Based Study. Cancers.

[55] Takeyama H., Noura S., Suzuki Y., Odagiri K., Yanagimoto Y., Yamashita M., Shimizu J., Kawase T., Imamura H., Iwazawa T. (2022). Higher Body Mass Index Is a Simple Favorable Non-Cancer Prognostic Marker for Japanese Elderly Colorectal Cancer Patients after Curative Resection. J. Anus Rectum Colon.

[56] An Y., Wu Z., Wang N., Yang Z., Li Y., Xu B., Sun M. (2020). Association between Body Mass Index and Survival Outcomes for Cancer Patients Treated with Immune Checkpoint Inhibitors: A Systematic Review and Meta-Analysis. J. Transl. Med..

[57] Ligibel J.A., Alfano C.M., Courneya K.S., Demark-Wahnefried W., Burger R.A., Chlebowski R.T., Fabian C.J., Gucalp A., Hershman D.L., Hudson M.M. (2014). American Society of Clinical Oncology Position Statement on Obesity and Cancer. J. Clin. Oncol..

[58] Mullen J.T., Davenport D.L., Hutter M.M., Hosokawa P.W., Henderson W.G., Khuri S.F., Moorman D.W. (2008). Impact of Body Mass Index on Perioperative Outcomes in Patients Undergoing Major Intra-Abdominal Cancer Surgery. Ann. Surg. Oncol..

[59] Wu X.-S., Wu W.-G., Li M.-L., Yang J.-H., Ding Q.-C., Zhang L., Mu J.-S., Gu J., Dong P., Lu J.-H. (2013). Impact of Being Overweight on the Surgical Outcomes of Patients with Gastric Cancer: A Meta-Analysis. World J. Gastroenterol..

[60] Kroenke C.H., Neugebauer R., Meyerhardt J., Prado C.M., Weltzien E., Kwan M.L., Xiao J., Caan B.J. (2016). Analysis of Body Mass Index and Mortality in Patients with Colorectal Cancer Using Causal Diagrams. JAMA Oncol..

[61] Mayr R., Fritsche H.-M., Zeman F., Reiffen M., Siebertz L., Niessen C., Pycha A., van Rhijn B.W.G., Burger M., Gierth M. (2018). Sarcopenia Predicts 90-Day Mortality and Postoperative Complications after Radical Cystectomy for Bladder Cancer. World J. Urol..

[62] Yang T., Luo K., Deng X., Xu L., Wang R., Ji P. (2022). Effect of Sarcopenia in Predicting Postoperative Mortality in Emergency Laparotomy: A Systematic Review and Meta-Analysis. World J. Emerg. Surg..

[63] Morshed R.A., Young J.S., Casey M., Wang E.J., Aghi M.K., Berger M.S., Hervey-Jumper S.L. (2022). Sarcopenia Diagnosed Using Masseter Muscle Diameter as a Survival Correlate in Elderly Patients with Glioblastoma. World Neurosurg..

[64] Berardi G., Antonelli G., Colasanti M., Meniconi R., Guglielmo N., Laurenzi A., Ferretti S., Levi Sandri G.B., Spagnoli A., Moschetta G. (2020). Association of Sarcopenia and Body Composition with Short-Term Outcomes after Liver Resection for Malignant Tumors. JAMA Surg..

[65] Meister F.A., Lurje G., Verhoeven S., Wiltberger G., Heij L., Liu W.-J., Jiang D., Bruners P., Lang S.A., Ulmer T.F. (2022). The Role of Sarcopenia and Myosteatosis in Short-and Long-Term Outcomes Following Curative-Intent Surgery for Hepatocellular Carcinoma in a European Cohort. Cancers.

[66] Wei K., Nyunt M.-S.-Z., Gao Q., Wee S.-L., Yap K.-B., Ng T.-P. (2018). Association of Frailty and Malnutrition With Long-Term Functional and Mortality Outcomes Among Community-Dwelling Older Adults: Results from the Singapore Longitudinal Aging Study 1. JAMA Netw. Open.

[67] Shamliyan T., Talley K.M.C., Ramakrishnan R., Kane R.L. (2013). Association of Frailty with Survival: A Systematic Literature Review. Ageing Res. Rev..

[68] Nguyen Q.D., Wu C., Odden M.C., Kim D.H. (2019). Multimorbidity Patterns, Frailty, and Survival in Community-Dwelling Older Adults. J. Gerontol. A Biol. Sci. Med. Sci..

[69] Zachariah F.J., Rossi L.A., Roberts L.M., Bosserman L.D. (2022). Prospective Comparison of Medical Oncologists and a Machine Learning Model to Predict 3-Month Mortality in Patients with Metastatic Solid Tumors. JAMA Netw. Open.

[70] D’Journo X.B., Boulate D., Fourdrain A., Loundou A., van Berge Henegouwen M.I., Gisbertz S.S., O’Neill J.R., Hoelscher A., Piessen G., van Lanschot J. (2021). Risk Prediction Model of 90-Day Mortality after Esophagectomy for Cancer. JAMA Surg..

